# Experimental Permeability and Porosity Determination of All-Oxide Ceramic Matrix Composite Material

**DOI:** 10.3390/ma17143612

**Published:** 2024-07-22

**Authors:** Ryszard Szwaba, Pawel Madejski, Piotr Kaczynski, Marcin Kurowski, Mathias Kunz, Katarzyna Berent, Tomasz Ochrymiuk

**Affiliations:** 1 Institute of Fluid-Flow Machinery, Polish Academy of Sciences (IMP PAN), Fiszera 14, 80-231 Gdansk, Poland; pkaczynski@imp.gda.pl (P.K.); mkurowski@imp.gda.pl (M.K.); tomasz.ochrymiuk@imp.gda.pl (T.O.); 2 Department of Power Systems and Environmental Protection Facilities, Faculty of Mechanical Engineering and Robotics, AGH University of Krakow (AGH), Adama Mickiewicza 30, 30-059 Krakow, Poland; 3 WPX Faserkeramik GmbH, Redcarstr. 44 B, D-53842 Troisdorf, Germany; mathias.kunz@whipox.com; 4 Academic Centre for Materials and Nanotechnology, AGH University of Krakow (AGH), Adama Mickiewicza 30, 30-059 Krakow, Poland; kberent@agh.edu.pl

**Keywords:** ceramic matrix composites, WHIPOX composite, water permeability, experimental measurements, scanning electron microscopy

## Abstract

This paper presents an investigation into the water permeability of an all-oxide ceramic matrix composite. To determine the parameters and characterize the water permeability of the ceramic composite material, an experimental study was carried out in which a dedicated test rig was constructed and commissioned. A total of five different configurations of composite tubes were tested. They differed in fibre roving strength, winding angle, fibre bundle arrangement during winding, and matrix grain size distribution. To better understand the internal structure of the analysed ceramic matrix composite material, the experimental study used scanning electron microscopy for microstructure and porosity observation. The tested tubes will be used as liners in an oxy-combustion chamber in future studies. The experiments obtained new and interesting results regarding the water permeability of the ceramic matrix composite with different structural parameters. It was also observed that, as with some porous materials, the permeability of ceramic matrix composites decreases with time as more and more liquid is pressed through it.

## 1. Introduction

Metallic materials, including superalloys, are near the upper-temperature limit of around 1100 °C required in today’s advanced machinery and equipment. As thermal load requirements increase, alternative materials, such as composite ceramics, are required to meet the demands of increasing operating temperatures. Advanced ceramic composites, in which the ceramic matrix is reinforced with continuous ceramic fibres, are characterized by low density, high strength and hardness at high temperatures, and excellent chemical stability. Ceramic matrix composites (CMCs) are at the forefront of advanced materials technology due to their high strength, high-temperature resistance, and relatively high resistance to damage under load. These properties are achieved by the correct design of the fibre–matrix interface, which helps to stop the propagation of cracks formed in the brittle matrix under load and prevents premature failure through fibre reinforcement.

Ceramic materials produced by CMC technology are currently used in a wide range of scientific and technical applications, but the authors’ particular interest is in applications in advanced aircraft engines, stationary gas turbines, heat exchangers, and highly thermally stressed combustion chambers. In these applications, materials that combine the high-temperature stability of ceramics with the nonbrittle fracture behaviour and thermal shock resistance typical of metallic arrangements are needed. Such properties are provided by the all-oxide, continuous fibre-reinforced ceramic matrix composite WHIPOX^®^ (Wound Highly Porous Oxide ceramic matrix composite), an original development of the Materials Research Institute of the German Aerospace Center (DLR), which is commercially available from the DLR spin-off WPX Faserkeramik GmbH (Troisdorf, Germany). Such CMC liners can increase the combustion temperature while reducing the need for coolant, thereby contributing to increased efficiency and reduced fuel consumption of an aircraft engine or gas turbine. Due to their excellent thermal cycling resistance and low heat capacity, WHIPOX components are ideally suited for operation at temperatures well above 1000 °C. The high permeability of WHIPOX makes it an attractive material for combustion chamber liners, which are exposed to extremely high temperatures and corrosion. In addition, this permeability can be controlled and designed to suit the requirements of the equipment by adjusting the manufacturing parameters. 

However, articles related to the study of permeability in CMC materials, such as WHIPOX, are virtually nonexistent in the literature. The publications that can be found cover a wide range of topics related to permeability in porous media, but few deal with ceramic matrix composites. They generally present the theoretical basis of flow through porous media, experimental techniques, modelling approaches, and practical applications. The studies emphasize the importance of pore-scale properties, fluid properties, and material structure in determining permeability behaviour. These publications provide valuable insights into fluid transport phenomena in porous materials and CMCs by combining theoretical concepts, experimental observations, and modelling methods.

The most common relationship used to describe permeability in porous materials is Darcy’s law [1,2,3], which expresses the relationship between fluid flow rate and pressure gradient in porous media and provides the basis for understanding permeability. More detailed relationships in fluid transport through porous media can be found in other works, including Dullien [4], who focuses on fluid transport and pore structure in porous media and discusses the mechanisms of fluid transport in porous media, emphasizing the importance of pore structure and pore size in determining permeability. In turn, the paper [5] analyses multiphase flow transport in porous media, considering factors such as interfacial tension, wettability, and the influence of pore geometry and fluid properties on flow patterns and permeability characteristics. Sahimi [6] also presents some flow and fluid transport aspects in fractured rock formations.

Reliable material data are extremely important for potential applications of a material such as WHIPOX, and these could be filters, thermal insulators or, in our case, combustion chamber liners. Some properties of WHIPOX have been reported in a handbook of ceramic composites [7], such as the thermal conductivity, which is anisotropic, i.e., the thermal conductivity in the direction of the fibres is about three times higher than in the direction perpendicular to the fibres. In this work, the air permeability of WHIPOX CMC was also determined to be very limited. The permeability is highly dependent on the processing parameters, matrix composition, and composite architecture. According to the publication, the flow rate increases with increasing roving strength, i.e., 1500 DEN (Denier) compared to 3000 DEN roving. In addition, higher airflow rates were obtained when a larger winding angle ±45° (intersection angle in 2D orientation) was used instead of fibre orientation ±15°. However, these results are difficult to apply to water permeability, as Darcy’s or Darcy–Forchheimer’s law does not consider all the fluid-related properties that affect the results of flow through porous materials, such as interfacial tension and wettability. The only publication that provides some data on water permeability through WHIPOX is the paper by Kirchberger et al. [8]. However, it does not provide any parameters for the WHIPOX sample tested, making it virtually impossible for others to use these data.

The WHIPOX materials that we studied are ceramic matrix composites (CMCs). Due to their exceptional properties, including high-temperature resistance, excellent thermal stability, and superior mechanical strength, they have garnered significant attention in advanced engineering applications [9,10]. Understanding the microstructural and mechanical properties of CMCs is crucial for optimizing their performance [11,12]. Determining the porosity of these materials is particularly important. Scanning electron microscopy (SEM) provides a powerful tool for investigating the microstructure and porosity of materials. Additionally, the SEM equipped with a focused ion beam (FIB) allows for precise surface preparation for observation, which offers high-resolution imaging capabilities that allow for the detailed examination of fibre–matrix interfaces, porosity, and crack propagation within the composites. 

Due to the very limited data available in the literature on the water permeability of WHIPOX material, it was decided to carry out an appropriate experimental study to determine its parameters and characterize the water permeability of a very advanced and promising ceramic composite material, WHIPOX. An additional novelty presented in the paper is the use of the FIB technique, which allowed us to obtain information about the size of the smallest pores, which was nanometric, and to determine the porosity of the analysed WHIPOX sample.

## 2. Materials and Experimental Setup

The WHIPOX composite material was developed by the German Aerospace Centre (DLR). The material consists of an oxide matrix (alumina–silicates or alumina) reinforced by oxide fibres (alumina–silicates or alumina). The matrix of these composites is highly porous, allowing damage tolerance without a specific fibre/matrix interphase. A simple winding process can produce oxide fibre/matrix composites, and the WHIPOX manufacturing technique allows a variety of component structures, sizes, and shapes to be produced by forming, joining, and machining. The basic manufacturing step is the design of the WHIPOX body and the corresponding optimized filament winding pattern. Prior to filament winding, the fibres are infiltrated with a liquid dispersion of the matrix particles and then wound onto a rotating mandrel. The wound bodies are dried and sintered at high temperatures to form a rigid CMC. The typical thickness of a single component ranges from less than 0.5 mm to more than 5 mm. In addition to the ceramic fibres used and the design of the winding pattern, the development of ceramic matrices is key to achieving optimized CMC material properties. Manufacturing uses high-end equipment to synthesize, prepare, and characterize ceramic matrix materials, such as in the production of the CMC material under study, the sintering temperature used was T = 1200 °C for 90 min.

**Figure 1:**
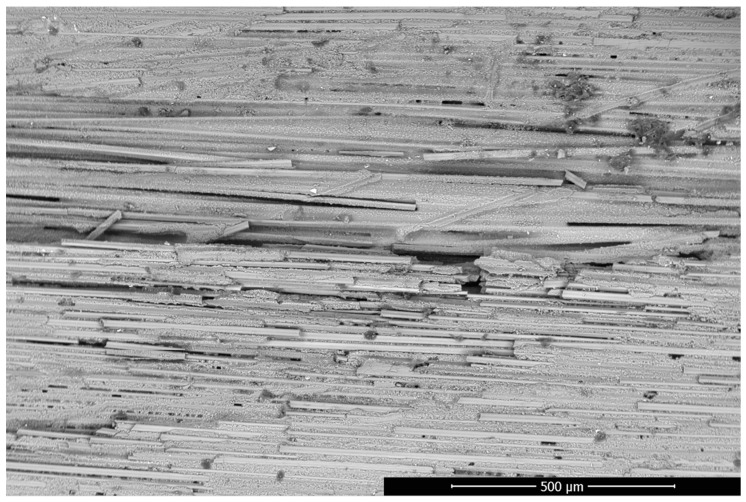
BSE/SEM (Back Scattered Electrons/Scanning Electron Microscope) image of fibres and matrix arrangement in the WHIPOX Ceramic Matrix Composite (CMC) sample.

In Figure 1, the WHIPOX fracture consisting of the matrix and the fibres can be observed. Solid lines represent fibres, and the material between them is the matrix made of the same material. Image analysis of WHIPOX material is possible thanks to a 3D scanning electron microscope (SEM). The irregularity of the fibre and matrix arrangement results from the fact that the presented image is the result of looking at the outer surface of the analysed sample.

**Figure 2:**
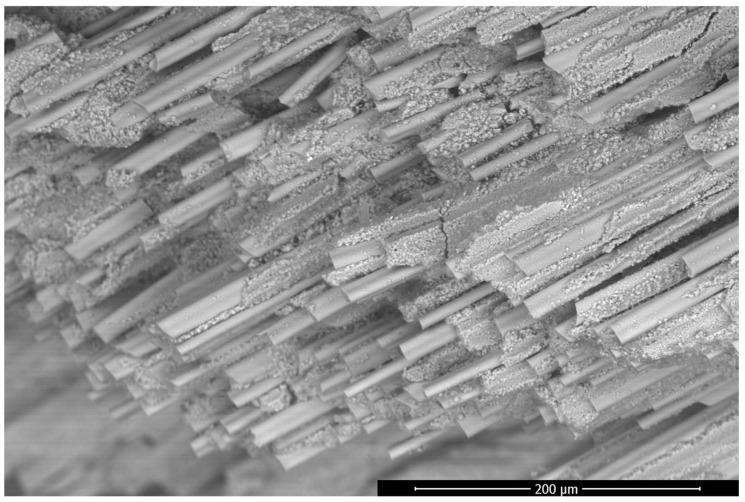
BSE/SEM (Backscattered Electrons/Scanning Electron Microscope) image of a fibre/matrix fracture showing the connection and distribution of fibres in the WHIPOX Ceramic Matrix Composite (CMC) sample.

In Figure 2, the connections between fibres and matrix are visible when the porous space is observable as open spaces in the matrix and gaps between the fibres and the matrix. The arrangement and parameters with which the fibres are wound may, therefore, affect the total porosity and permeability of the sample, although the authors assumed that porous space in the matrix itself is similar and can be computed using FIB-SEM results analysis.

In the study presented in this article, five samples produced by WPX Faserkeramik GmbH with different production parameters were tested. They differed in the fibre roving strength, the winding angle, the arrangement of the fibre bundles during winding, and the grain size distribution of the matrix. A list of the samples used and their parameters is given in Table 1.

**Figure 3:**
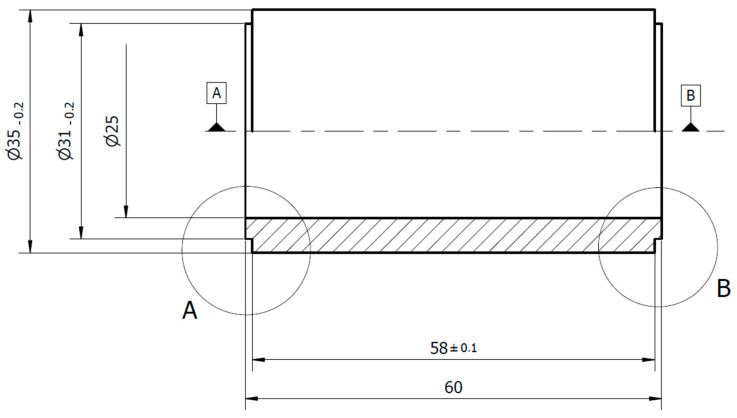

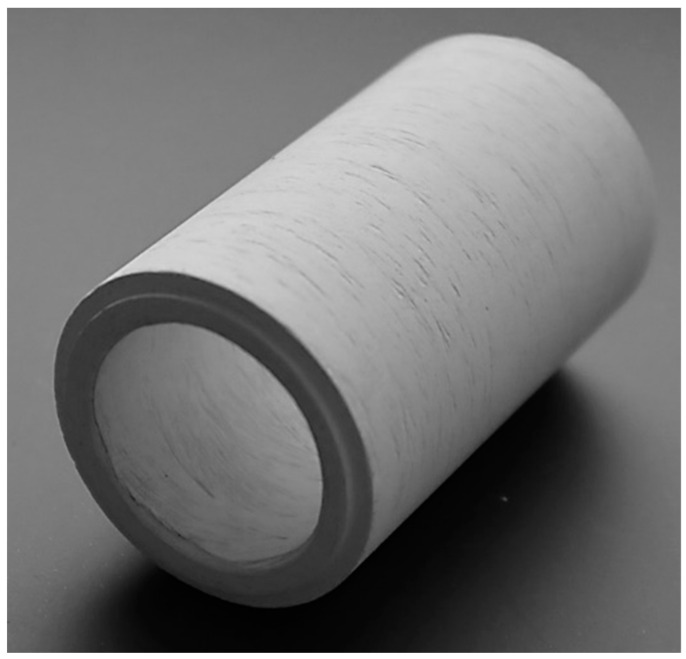
WHIPOX tube, top-design drawing, down-picture view.

As can be seen, the basic parameters of the WHIPOX tube structure, such as roving and winding angle, were in the range of 1500 to 20,000 DEN and ±10 and ±30°, respectively. The fibre material is commercially available 3M Nextel 610 (Al_2_O_3_). Figure 3 below shows the dimensions of the WHIPOX tube to be tested and a photo of the tube already manufactured by WPX Faserkeramik GmbH. The notches visible on the left and right outer diameters are intended to receive the O-ring seal from the front of the tested tube. According to the dimensions given, the internal surface area of the tube through which the water is forced is 47.1 square centimetres.

**Figure 4:**
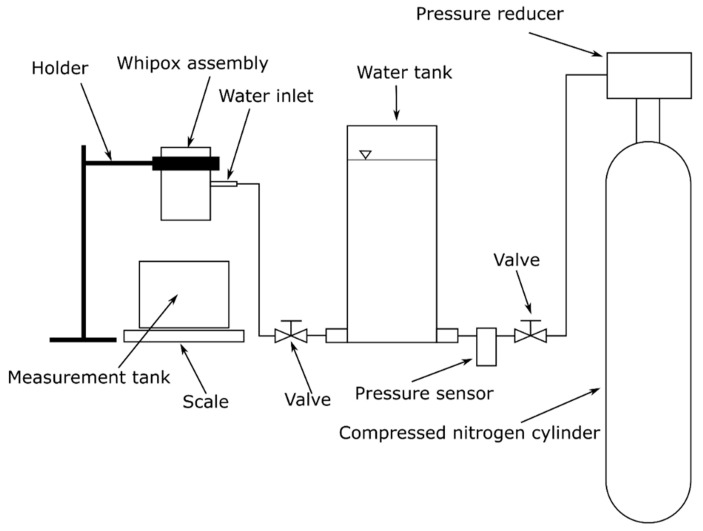
Scheme of the test stand for WHIPOX permeability tests.

A schematic diagram of the test rig for measuring water permeability through a porous WHIPOX ceramic composite material is shown in Figure 4. The operating principle of the test system is the following: gas from a cylinder filled with compressed nitrogen is directed into a water tank. The operating pressure is controlled by an automatic pressure control valve located at the cylinder outlet, and its function is to maintain a constant pressure level throughout the measurement. There is also a shut-off valve and a pressure sensor between the water tank and the cylinder. Compressed nitrogen at the set pressure forces water out of the tank, which enters the WHIPOX assembly through the water connection. The gas in the tank is separated from the water by an impermeable membrane to prevent the gas from dissolving in the water under pressure.

The water connection is connected to a threaded cylinder in which the WHIPOX porous element is embedded. The WHIPOX is supported at its ends by reduction sleeves screwed into the threaded cylinder. This design causes the water to enter the threaded cylinder and then be squeezed through the walls of the porous element into its inner wall, which then flows into the measuring tank. In the permeability tests, demineralised water was used. The mass flow rate is calculated by measuring the weight of water squeezed through the WHIPOX and entering the water tank after a certain time. A computer timer automatically measured the time, while the water weight was measured using a laboratory balance with a range of 0–2000 g, a reading accuracy of 1 mg, and an overall accuracy of ±0.1 g.

## 3. Matrix Porosity Determination Using FIB-SEM Tomography

Microstructural studies of the WHIPOX material were performed using an FEI Versa 3D scanning electron microscope (SEM) (FEI, Hillsboro, OR, USA) equipped with a secondary (SE) and backscattered electron (BSE) detector at an accelerating voltage of 15 kV. SEM observations were made of the fracture, and a cross section of the sample was prepared using the focused ion beam (FIB) technique. The cross section was prepared at a Ga+ ion beam current of 3 nA and a beam energy of 30 kV.

**Figure 5:**
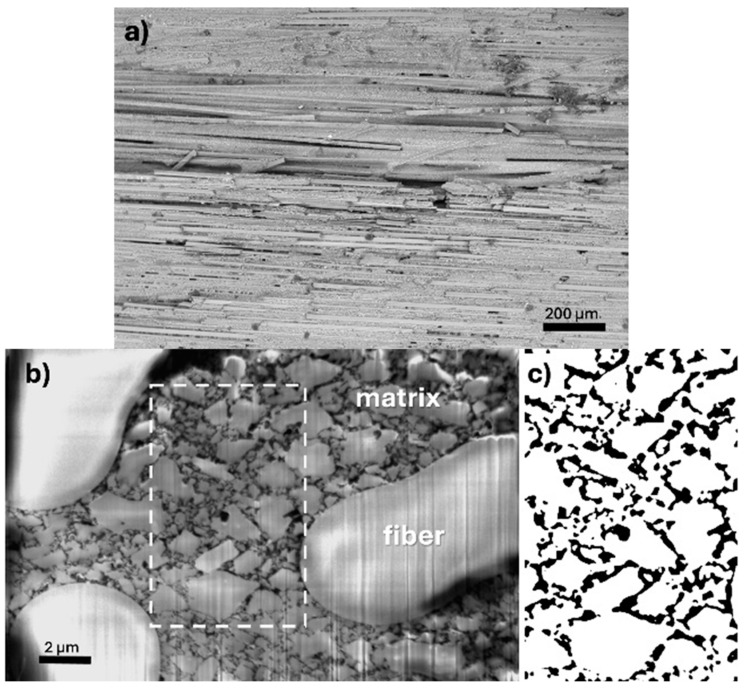
SEM image of the fracture surface (**a**), SEM image of the cross section (**b**), and the corresponding binary converted SEM image of the WHIPOX matrix (**c**).

WHIPOX is an example of a ceramic matrix composite (CMC). Figure 5a shows the WHIPOX fracture. Both the matrix and the fibres can be distinguished. Continuous long fibres embedded in a fine-grained matrix are observed (Figure 5b). The matrix presents homogeneously distributed crystalline phases, with a porosity of 24.2% calculated from the proportion of black (pores) to white (ceramic) areas in the binary image (Figure 5c). The porosity was calculated from the WHIPOX matrix using ImageJ [13] software, which converts an SEM image to a binary image and thresholds it. For analysis and porosity calculations, the test sample was divided into 800 slices and exported as tiff images to ImageJ program.

## 4. Results and Discussions

**Figure 6:**
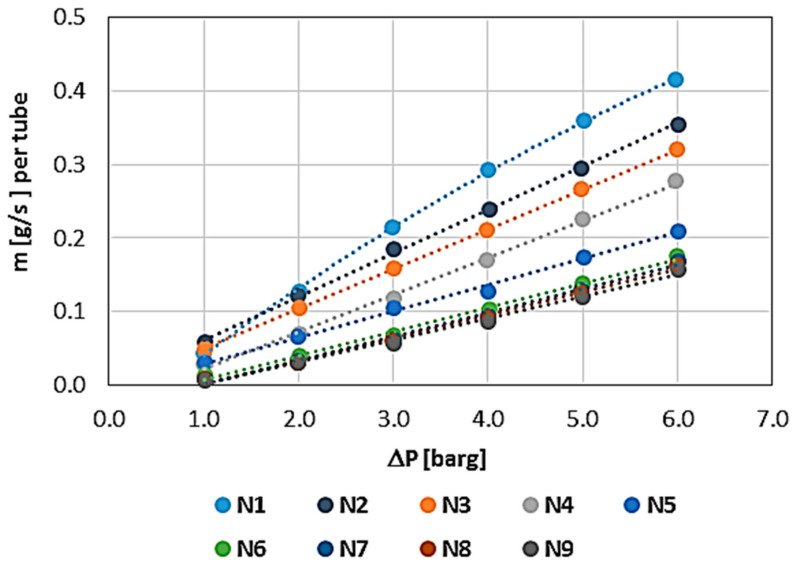
WHIPOX permeability test for T1 tube.

**Figure 7:**
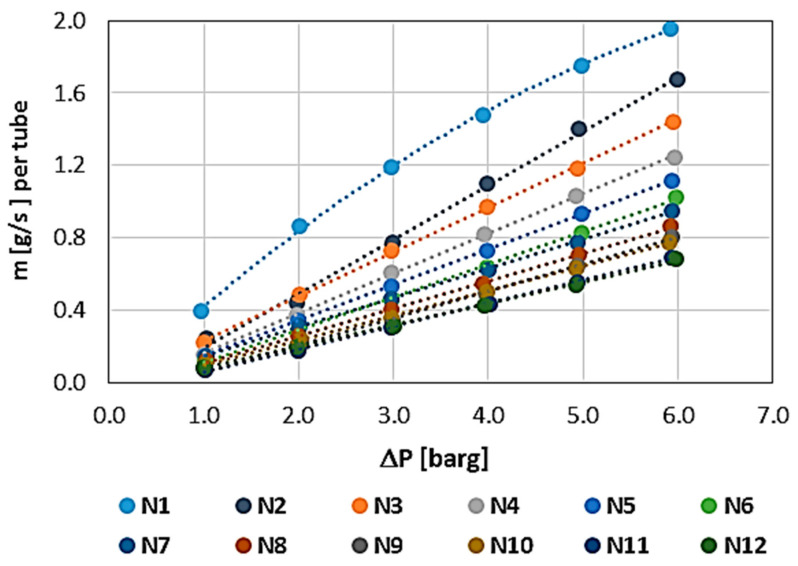
WHIPOX permeability test for T2 tube.

**Figure 8:**
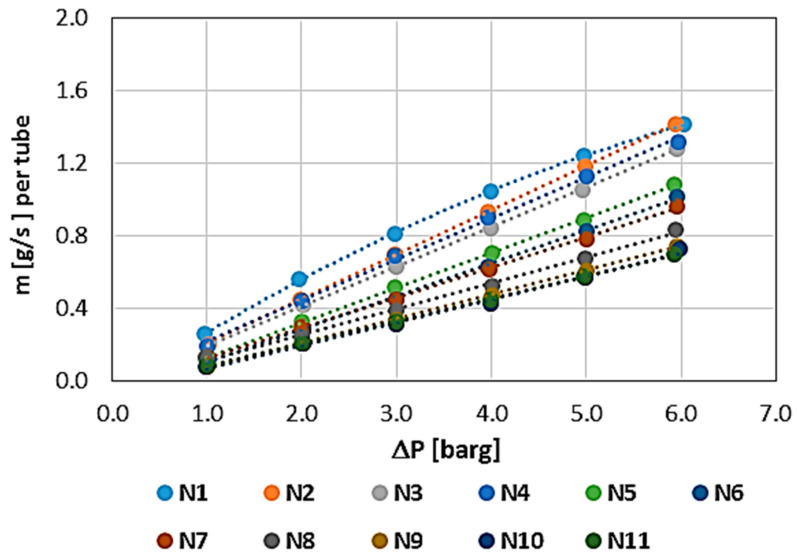
WHIPOX permeability test for T3 tube.

**Figure 9:**
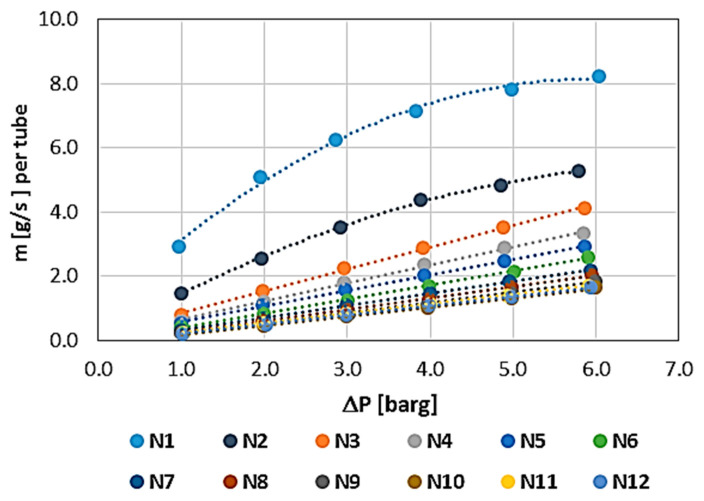
WHIPOX permeability test for T4 tube.

**Figure 10:**
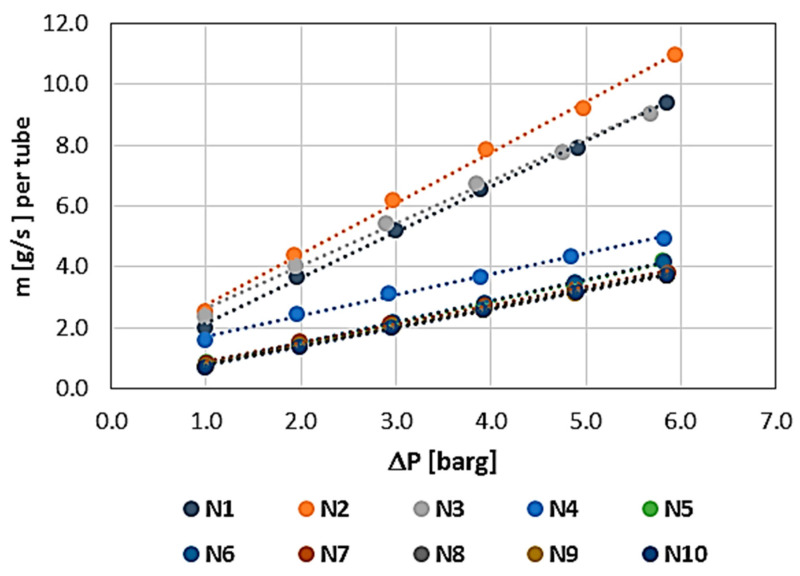
WHIPOX permeability test for T5 tube.

The results showing the permeability characteristics for individual WHIPOX tubes (T1–T5) are shown in Figure 6, Figure 7, Figure 8, Figure 9 and Figure 10. For each tube, the mass flow rate is shown as a function of the pressure difference on either side of the tube wall, i.e., between the outside and inside diameters. The reference pressure in these measurements is the ambient pressure [barg—gauge pressure] on the inside of the tube. The outside was the pressure side. In these plots, the mass flow is related to the permeability of the whole tube, which is convenient in the first approach since, in further applications of the tested tubes, it is necessary to know the permeability of a single tube. In the rest of the article, the permeability values are normalized.

All the tubes measured were brand new, i.e., the first characteristic corresponds to the permeability of a dry tube, requiring only air release, i.e., the removal of gas from the inside of the wall. The method of measuring each characteristic was the same and proceeded sequentially from the lowest pressure (1 bar) to the maximum pressure (approximately 6 bar). The mass flow measurement time at a given point, i.e., at a constant, fixed pressure, was 180 s (3 min). Each successive characteristic was measured in the same way.

The first measured characteristic for a given tube was marked with the number N1 (blue), while subsequent numbers from N correspond to the next characteristic in the measurement series for each WHIPOX tube. As can be seen from Figure 6, Figure 7, Figure 8, Figure 9 and Figure 10, several different characteristics were obtained for each WHIPOX tube tested. The highest permeability values were obtained for the first characteristic in the series (light blue colour). As successive series are measured, a given tube’s permeability (mass flow) gradually decreases. After about 10 series, the output, which may be more or less depending on the tube parameters, begins to stabilize. If the average mass flow rate for a given characteristic did not differ by more than 1% from the previous one, the measurement of the next characteristic was not continued, and it was considered that such a characteristic would already determine the permeability of a given WHIPOX tube in the long term.

Figure 6, Figure 7, Figure 8, Figure 9 and Figure 10 show that the first curves in the series have a nonlinear characteristic (high flow rates), and only the subsequent lines become linear as the mass flow decreases. At low flow rates, Darcy’s law correctly describes flow in porous media [14]. However, as the velocity increases, there is a discrepancy between the experimental data and the results obtained for Darcy’s law. Forchheimer related this discrepancy to inertial effects and suggested adding a term representing kinetic energy to the expression [15,16].

The decrease in the permeability of porous materials over time, as more and more liquid is forced through them, is a well-known phenomenon, but WHIPOX is just such a material made of composite ceramics. The phenomenon relates to injecting liquid under pressure into the small pores and creating a thin film of irreducible liquid saturation around the pores and within the pore channels. Irreducible saturation is maintained by surface tension and often by high capillary pressure [17,18,19]. Irreducible water saturation can be estimated from capillary pressure data or nuclear magnetic resonance spectroscopy. Irreducible water saturation occurs due to internal forces between liquid and pore walls, especially in nano- and micropores. In general, it can be one of the effects that reduce the permeability in the analysed material. 

Authors [20] investigated the permeability coefficient of samples with different initial water content and dry density conditions. The results confirmed that permeability decreases with increasing dry density. The trend of permeability changes was observed for all the samples analysed, and the seepage time varies between samples. The results indicated that permeability has a good negative exponential relationship with dry density during infiltration. Dry density is a parameter that directly reflects the influence of the compactness of the pore structure on the permeability, which is determined by the skeletal structure in the analysed sample. This phenomenon can explain different permeability changes for different samples.

**Figure 11:**
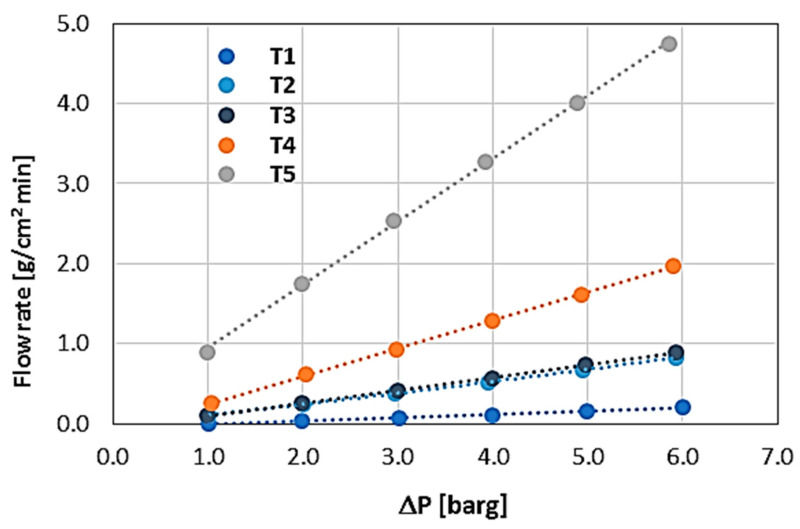
WHIPOX permeability normalised results for all tubes, T1–T5.

Figure 11 shows the normalized mass flow characteristics for each WHIPOX tube. As shown in the figure, the output unit, in this case, is [g/cm^2^·min]. The size of the inner surface of the tube was used for normalisation. From the analysis of the tests shown in Figure 11, it can be seen that the main parameter affecting the permeability of the tube wall is its roving strength. The lower the roving strength, the lower the permeability. This is an expected result as this parameter determines the weight of a unit length of fibre (9000 m). A thinner roving means less weight, which in turn means less roving strength. A thinner roving means less porosity in the production of a given composite ceramic sample. Of course, permeability is also influenced by the winding angle of the fibre. A larger winding angle promotes higher permeability. The higher permeability for larger winding angles is due to the fact that the angle formed by fibre bundles winding in opposite directions increases, and thus, in combination with the matrix, the porosity of the composite material also increases.

In addition, T2 and T3 tubes have the same roving (10,000 DEN) and winding angle (+/−30°), although they differ in one parameter, namely the so-called FBD (fibre bundle displacement). As can be seen in Figure 11, this parameter has little effect on the permeability of the material as WHIPOX, and in this case, the average mass flow increases by approximately 5%. Tubes T4 and T5 also have the same roving (20,000 DEN) and winding angle (+/−30°), while they differ in the filament winding parameters. However, changing this parameter significantly affects the tube’s permeability in this case. By changing the overlap of the laid fibres in certain areas, it is possible to increase the mass flow by approximately 2.5 times (see T5 vs. T4).

The permeability characteristics shown in Figure 11 are linear, so Darcy’s law, which contains only one linear term, is sufficient for their general description. In general, Darcy’s law can be expressed as:

$$\frac{dp}{dz}=\frac{\eta }{k_{D}}v$$

where: *dp*—pressure difference, *dz*—thickness of the tube wall, *η*—dynamic viscosity, *ν*—volume flux vector or filtration velocity, *k_D_*—permeability coefficient.

**Figure 12:**
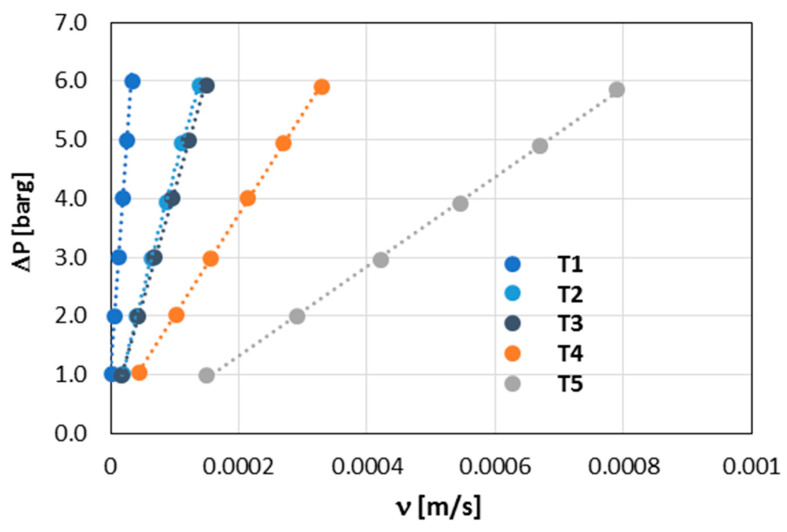
Normalized WHIPOX permeability results in terms of Darcy’s law for all tubes, T1–T5.

The same data shown in Figure 11 can be plotted as the velocity filtration vector versus pressure drop (see Figure 12). This provides a direct route to the already more general permeability coefficient *k_D_*. These coefficients, determined for each WHIPOX tube (T1–T5), are given in Table 2.

As can be seen in Table 2, the permeability coefficients of WHIPOX tubes for rovings 10,000 and 20,000 (T2–T5) are of the order of 10^−10^ and are in the range of 1.31 × 10^−10^–7.08 × 10^−10^. In turn, the permeability coefficient for tubes with the smallest roving is about one order lower. Darcy’s law shows that the *k_D_* coefficient is proportional to the mass flow of water through the tube wall, and the smaller the coefficient, the lower the porosity and permeability of the material through which the liquid is pressed.

## 5. Conclusions

Due to the very limited data available in the literature on the water permeability of WHIPOX all-oxide ceramic matrix composites, it was decided to carry out an appropriate experimental study to determine its parameters and characterise the water permeability of a very advanced and promising ceramic composite material in applications. For this purpose, a dedicated test rig was built and commissioned. The test element, manufactured using WHIPOX ceramic composite technology, was a 60 × 35 × 5 tube (length, outer diameter, wall thickness). The tested tubes will be used as liners in a pure oxygen combustion chamber in future studies. In total, five different configurations of WHIPOX tubes were tested. They differed in fibre roving strength, winding angle, fibre bundle arrangement during winding, and matrix grain size distribution. The WHIPOX wall permeability measurement studies presented in this paper showed that:

- The highest permeability values were obtained for the first curve in the series. These characteristics are nonlinear. As successive series are measured, a given tube’s permeability (mass flow) gradually decreases. After about 10 series, the output, which may be more or less depending on the tube parameters, begins to stabilize, and the subsequent distributions become linear as the mass flow decreases. For these characteristics, Darcy’s law correctly describes the flow in a porous WHIPOX wall.
- The decrease in permeability over time in the WHIPOX material is related to the injection of liquid under pressure into the small pores, creating a thin film of irreducible liquid saturation around the pores and inside the pore channels.
- The lower the roving strength, the lower the permeability. Permeability is also affected by the winding angle of the fibre. A larger winding angle promotes higher permeability, but ultimately, a larger winding angle with all other parameters remaining the same will result in increased porosity.
- The arrangement of the fibre bundles during winding at the same roving and winding angle has no significant effect on the permeability (about 5%). In contrast, the filament winding parameter for the same roving and winding angle significantly affects the WHIPOX wall’s permeability. Changing the overlapping of the laid fibres in certain areas can increase the permeability by at least two times.
- The permeability coefficients of WHIPOX tubes for 10,000 and 20,000 rovings are of the order of 10-10 and are in the range of 1.31 × 10^−10^–7.08 × 10^−10^. In turn, the permeability coefficient for the tube with the smallest roving is about one order lower.
- Thanks to the FEI Versa 3D scanning electron microscope (SEM), the porosity of the matrix material sample was calculated. Even if the porosity of the tested sample is high, around 24.2%, the parameters of specimens used in the experiment have a dominant impact on the measured permeability values.

WHIPOX CMC materials are versatile and valuable across various industries due to their high-temperature resistance, low weight, and excellent mechanical properties. In aerospace, they enhance engine components and thermal protection systems for spacecraft. The automotive industry benefits from their application in high-performance brake systems and exhaust components. In the energy sector, CMCs improve efficiency and durability in gas turbines, combustion chambers, and nuclear reactor components. Industrial applications include their use in heat exchangers and furnace components, while in defence, they are used in missile nose cones and armour and many others similar applications.

## Figures and Tables

**Table 1:** Parameters of specimens used in the experiment.

Sample Designation	Roving [DEN *]	Winding Angle [°]	Fiber Bundle Displacement [mm]	Filament Winding Parameters	Matrix Grain Size Distribution
T1	1500	±10	0	-	System 1
T2	10,000	±30	0	-	System 1
T3	10,000	±30	3	-	System 1
T4	20,000	±30	0	4 × 110%	System 2
T5	20,000	±30	0	110% + 2 × 100% + 110%	System 2

* DEN (Denier)—unit of measurement of the mass of linear density of the yarns. A fibre with a length of 9000 metres and a mass of 1 g has a density of 1 denier.

**Table 2:** Permeability coefficients.

Sample Designation	Roving [DEN]	Winding Angle [+/− °]	Permeability Coefficient *k_D_*, [m^2^]
T1	1500	10	3.45 × 10^−11^
T2	10,000	30	1.31 × 10^−10^
T3	10,000	30	1.43 × 10^−10^
T4	20,000	30	3.14 × 10^−10^
T5	20,000	30	7.08 × 10^−10^

## Data Availability

The original contributions presented in the study are included in the article, further inquiries can be directed to the corresponding authors.
